# Lymphological Liposculpture for Secondary Lymphedema after Breast Cancer and Gynecological Tumors: Long-Term Results after 15 Years

**DOI:** 10.1055/s-0043-1768943

**Published:** 2023-05-29

**Authors:** Manuel E. Cornely

**Affiliations:** 1 LY.SEARCH, Cologne, North Rhine-Westphalia, Germany

**Keywords:** lymphedema, complex decongestive therapy, mammary carcinoma, cervical carcinoma, liposuction, lipohyperplasia dolorosa

## Abstract

**Background** Untreated lymphedema of an extremity leads to an increase in volume. The therapy of this condition can be conservative or surgical.

**Methods** “Lymphological liposculpture” is a two-part procedure consisting of resection and conservative follow-up treatment to achieve curative volume adjustment of the extremities in secondary lymphedema. This treatment significantly reduces the need for complex decongestive therapy (CDT). From 2005 to 2020, 3,184 patients with secondary lymphedema after breast cancer and gynecological tumors were treated in our practice and clinic. “Lymphological liposculpture” was applied to 65 patients, and the data were recorded and evaluated by means of perometry and questionnaires.

**Results** The alignment of the sick to the healthy side was achieved in all patients. In 58.42% ( *n* = 38), the CDT treatment could be completely stopped postoperatively; in another 33.82% ( *n* = 22) of the patients, a permanent reduction of the CDT was achieved. In 7.69% ( *n* = 5) patients, the postoperative CDT could not be reduced. A total of 92.30% ( *n* = 60) of the patients described a lasting significant improvement in their quality of life.

**Conclusion** “Lymphological liposculpture” is a standardized curative sustainable procedure for secondary lymphedema for volume adjustment of the extremities and reduction of postoperative CDT with eminent improvement of the quality of life.

## Introduction

“Lymphedema is a hidden epidemic. It is one of the least recognized illnesses, among both doctors and patients.” 1

**Fig. 1:**
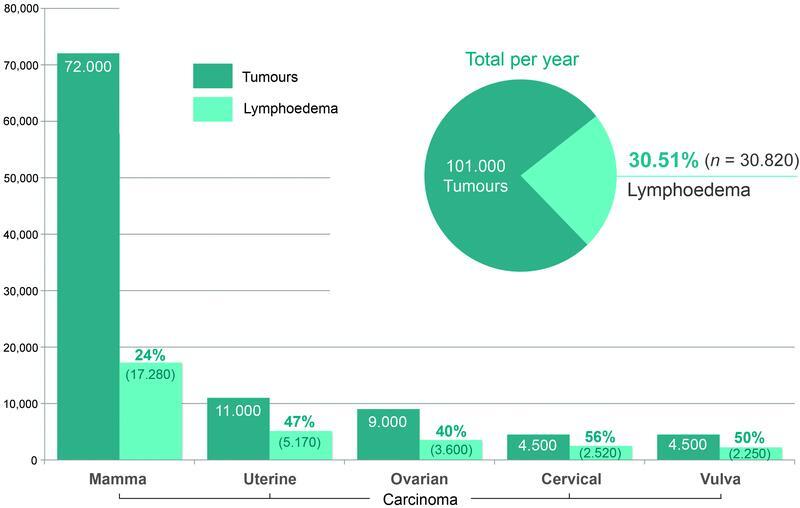
Incidence of secondary lymphedema after breast cancer or gynecological tumors per year in Germany.

In Germany, up to 101,000 patients are diagnosed with the tumors investigated in this study every year. Due to the disease, a lymph node dissection is usually performed as part of the oncological treatment. The incidence 2 of secondary lymphedema is between 24 and 70%, depending on the tumor. 3 4 In Germany, this corresponds to approximately 31,000 (30.51%) patients per year with this new lymphological condition caused by oncological treatment. It develops after oncological therapy in mammary carcinoma (24%), 3 uterine carcinoma (47%), 5 6 7 ovarian carcinoma (40%), 8 cervical carcinoma (56%), and vulva carcinoma (30–70%; Fig. 1 ).

The lymphatic vessels themselves are not initially damaged in secondary lymphedema after oncological treatment. 9 10 New development and growth of solid tissue in lymphedema in International Society of Lymphology (ISL) stages II and III 11 are, however, promoted by the surgical intervention on the lymphonodal system (lymphadenectomy) in the center of the limb. 12 Radiation and certain chemotherapeutic agents are also suspected of causing lymphedema and the subsequent tissue increase. 13 14 15 16 Other risk factors such as obesity also promote the development of lymphedema. 2 17 18

The increase in volume in lymphedema is not only relevant from a cosmetic point of view, but also from an orthopaedic point of view due to its weight. Movement restrictions can be a consequence of lymphedema. 11 19 20 21 22 23

**Fig. 2:**
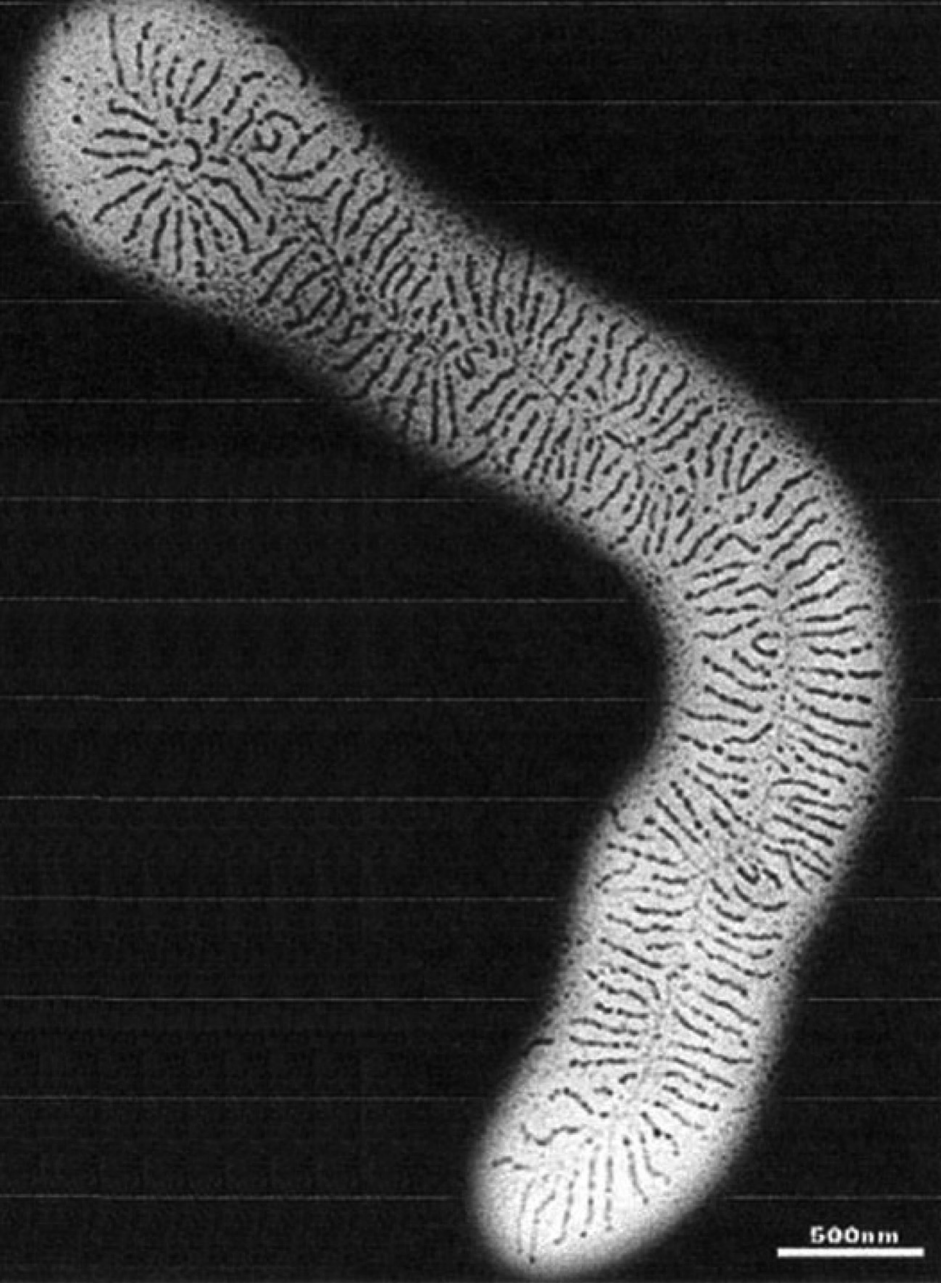
Hyaluronic acid keel and the protein central filaments. One can see the hyaluronic acid keel and the protein central filament. The glycosaminoglycans (50 keratan sulphates, chondroitin sulfate, dermatan sulfate, or heparan sulfate) are not visible. The “white space” is the area where water can be stored.

Although it is a scientifically outdated concept, the current treatment of choice for incipient lymphedema is still lifelong complex decongestive therapy (CDT) with manual lymphatic drainage (MLD), consistent compression with flat-knit compression stockings, skin care, and exercise therapy. 11 19 24 25 26 However, a paradigm shift is emerging. Vascular reconstruction by microsurgery, lymph node transplantation, and resection of the subcutaneous matrix are gaining importance in the treatment of secondary lymphedema. 27 28 29 30 31 32 33 34 35 36 37 38 If treatment is too late or inadequate, there is an excessive accumulation of interstitial proteoglycans (PG) and glycosaminoglycans (GAG) in the affected areas or extremities, which are supposed to bind the increasing fluid as a gel 12 39 40 41 ( Fig. 2 ).

As this process progresses, more PG and GAG are produced than degraded, leading to further proliferation of the volume. 9 12 39 42

Since the early 1960s, surgical procedures have been developed to improve the condition of secondary lymphedema. These include the development of the lymphovenous anastomosis (LVA) by Jacobson and Suarez, 43 its further development by Yamada 44 and later by Koshima et al, 45 the introduction of the lymphatic vessel transplantation (LVT) and the lympholymphatic bypasses (LVB) according to Baumeister et al, 46 the vascularized lymph node transfer (VLNT) developed by Becker et al, 47 the liposuction described by Brorson and Svensson, 48 and the “lymphological liposculpture” by Cornely. 36

The “lymphological liposculpture” procedure consists of two parts and includes a resecting, suctioning operation, as well as obligatory special follow-up treatment. It has been developed and standardized over the past 25 years from the experience of more than 13,000 procedures in lipohyperplasia dolorosa (LiDo) and in secondary lymphedema. 49 50 Postoperative examinations were performed regularly at 6 weeks, 6 months, and 1 year after surgery during the first year.

The goals of this surgical therapy for lymphedema are to reduce the volume, equalize the extremities, reduce the need for CDT, and improve the patients' quality of life. 2 42

This is the first study to retrospectively examine the long-term results of patients with secondary lymphedema who have undergone “lymphological liposculpture” as a therapy.

## Methods

In a retrospective analysis, 65 patients who were treated with “lymphological liposculpture” for their secondary lymphedema of ISL stages II ( *n* = 13) and III ( *n* = 52) after breast carcinoma or gynecological tumor between 2005 and 2020 were evaluated. 11 At that time, the diagnosis was made solely on the basis of history, inspection, and palpation. No instrumental verification of the clinically obvious lymphedema was performed. Also, no preoperative volume measurements by perometry were performed. The age of the patients at the time of the cancer, the type of cancer, the duration, and stage of the lymphedema, as well as the extent of conservative therapy (CDT) before and after “lymphological liposculpture” were evaluated.

**Fig. 3:**
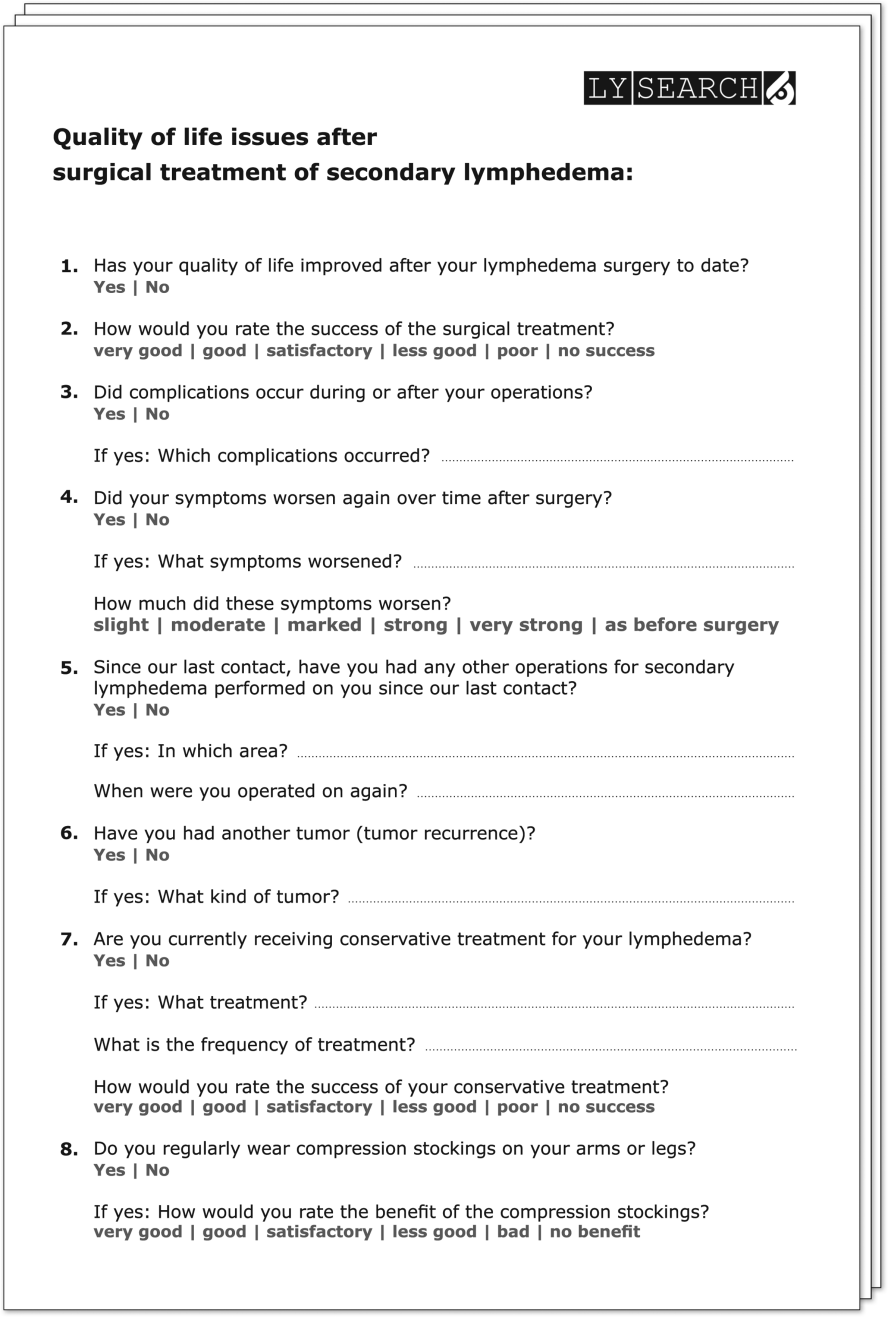
Quality of life questionnaire ( https://www.lysearch.de/home/evaluation-op-sek-lymphoedem ).

A self-designed 25-item Quality of Life Questionnaire (QoL) was used to collect data on changes in lymphedema, quality of life, and treatment success up to 15 years after resection surgery 51 ( Fig. 3 ).

QoL was collected in writing or by telephone in all 65 patients. Postoperative volumes were measured perometrically and the healthy versus operated limb of each patient was assessed. A comparison of the pre- and postoperative volumes of the operated limb was not planned. As not all patients could travel to the Ly.Search study center in Cologne, only 42 of the 65 participants could be measured.

This study was approved by the Institutional Review Board (IRB) with an approval number of 324/2022.

### The Two-part “Lymphological Liposculpture” Treatment Method

“Lymphological liposculpture” comprises an operative part as power-assisted liposuction (PAL) under tumescent local anesthesia with the addition of hyaluronidase (TLA + H) and an obligatory conservative follow-up treatment in a special setting as “accentuated manual lymphatic drainage (AMLD).” 2 9 42 50

In the case of arm lymphedema, surgery of the affected limb is performed in one session; in the case of secondary lymphedema of the legs following gynecological tumors, surgery is performed in two sessions, first on the lateral and 4 weeks later on the medial side of the leg(s). On the arm, the stab incisions for access to liposculpture are made in a radial position dorsally and ventrally at the wrist, ulnarly and radially at the elbow, and on the upper arm dorsally near the posterior axillary fold. For the outer side of the leg, the stab incisions are placed medially below the groin, medially on the mid-thigh, laterally on the mid-lower leg and on the lateral ankle. When operating on the inner side of the leg, the incisions of the first operation on the thigh are reopened, and a small incision is also made medially on the lower leg and on the medial ankle.

The adapted tumescent solution (TLA) according to Klein, 52 modified by the addition of 3,000 IU of the enzyme hyaluronidase (HYLASE “Dessau” 300 I.E., RIEMSER Pharma GmbH, Greifswald, Germany), per 500 mL (TLA + H) has a lytic effect on the central hyaluronan (HA) filament of the PG and supports the preparation of the subcutaneous matrix in lymphedema for the performance of lymph vessel–saving “lymphological liposculpture” ( Table 1 ). 37 This application of hyaluronidase is an off-label use.

**Table 1:** Formulation of tumescent local anesthesia with the addition of hyaluronidase

Component	Dose and concentration
Hyaluronidase	6,000 IU
Prilocaine	10 mL (1% without adrenaline)
Lidocaine	10 mL (2%)
Adrenaline	1 mL (1:1.000)
Sodium bicarbonate	10 mL (8.4%)
Triamcinolone	1 mL (40 mg)
NaCl 0.9%	1,000 mL

**Fig. 4:**
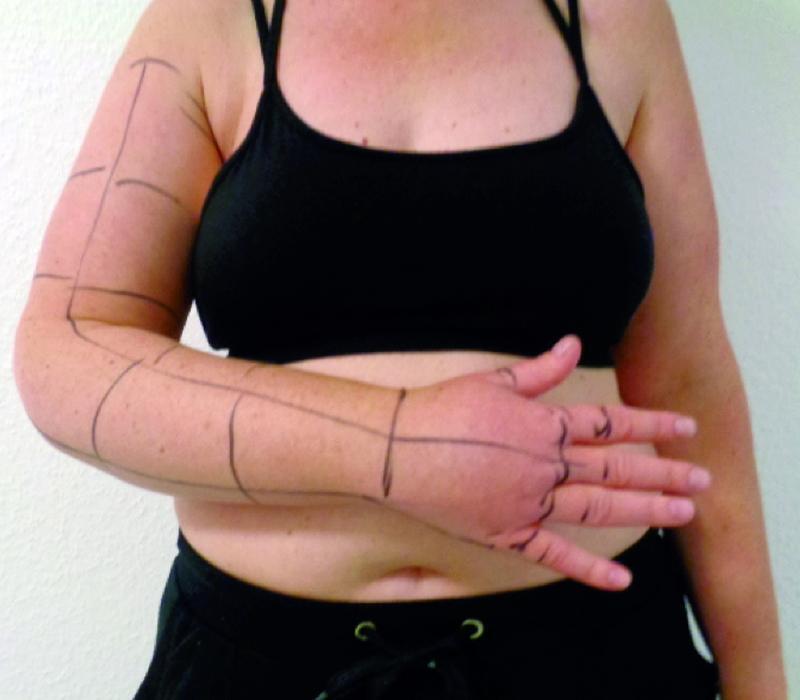
Preparation phase (tumescent local anesthesia with the addition of hyaluronidase).

The exposure time of TLA + H, i.e., the time between the application of this special TLA and the start of the “lymphological liposculpture” should be between 90 and 120 minutes as a tissue preparation phase. For tumescence and surgery for unilateral lymphedema of the leg, a total of up to 150 minutes should be allowed, for the treatment of both legs up to 180 minutes (outside or inside each in one session). For the operation of a lymphedema arm, 30 minutes are required after the preparation phase 53 ( Fig. 4 ). We deliver the TLA + H into the tissue via a pump with a flow of 300 mL/min. For arm surgery, between 1,000 and 2,000 mL of TLA + H is required to achieve a parallel elastic turgor of the tissue. This is usually accomplished on the sides of the legs, whether internal or external, with 3,000 to 4,000 mL. The upper limit for infiltration volume is set in our safety protocol at 10,000 mL per operation.

**Fig. 5:**
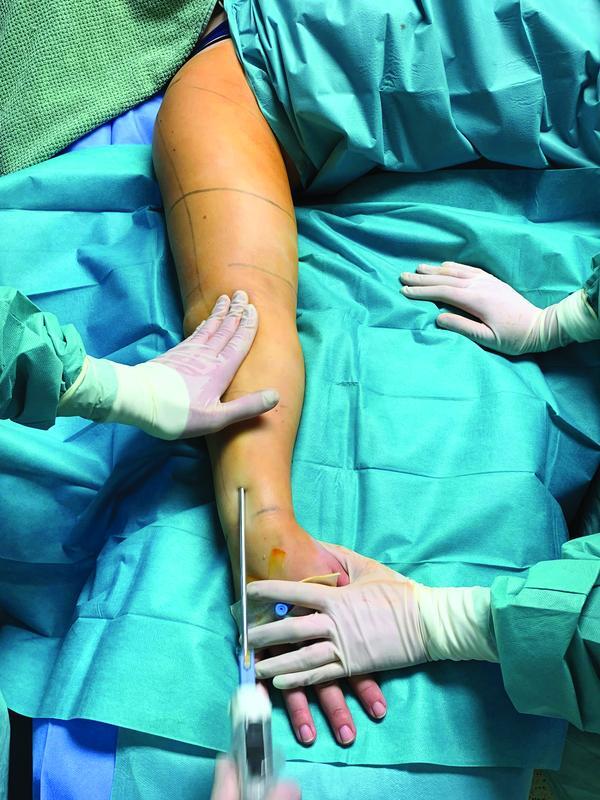
Strict longitudinal axial suction.

The operation is performed power-assisted under modified TLA with accompanying analgesia. For reasons of better handling for the patient and the surgeon, infiltration and “lymphological liposculpture” are always performed in complete analgesia with the assistance of the anesthesiologist, who usually performs the short switch on/switch off anesthesia with remifentanil (Ultiva) and propofol. The subcutaneous tissue is removed with a 5-mm cannula in vibration mode (PAL + TLA + H). Except for a superficial subcutaneous residuum of 10 mm, the tissue is aspirated longitudinally and axially, strictly avoiding cross and transverse maneuvers ( Fig. 5 ).

**Fig. 6:**
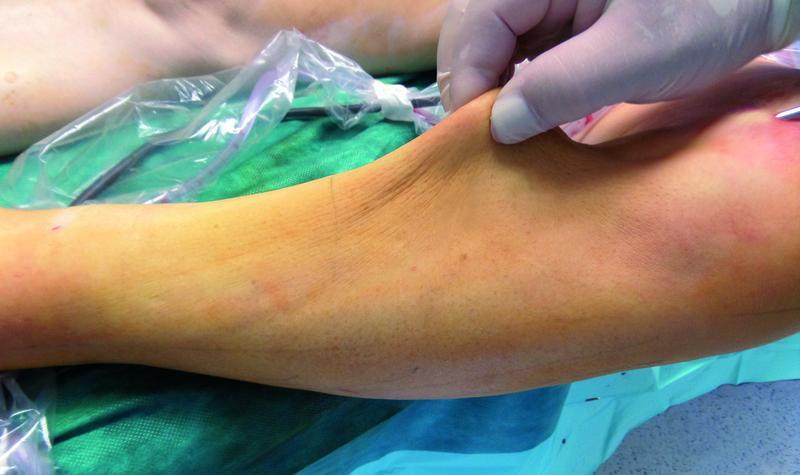
Skinfold thickness.

The thickening of the cutis (skinfold thickness [SFT]) itself, typical of lymphedema, cannot be thinned out ( Fig. 6 ).

**Fig. 7:**
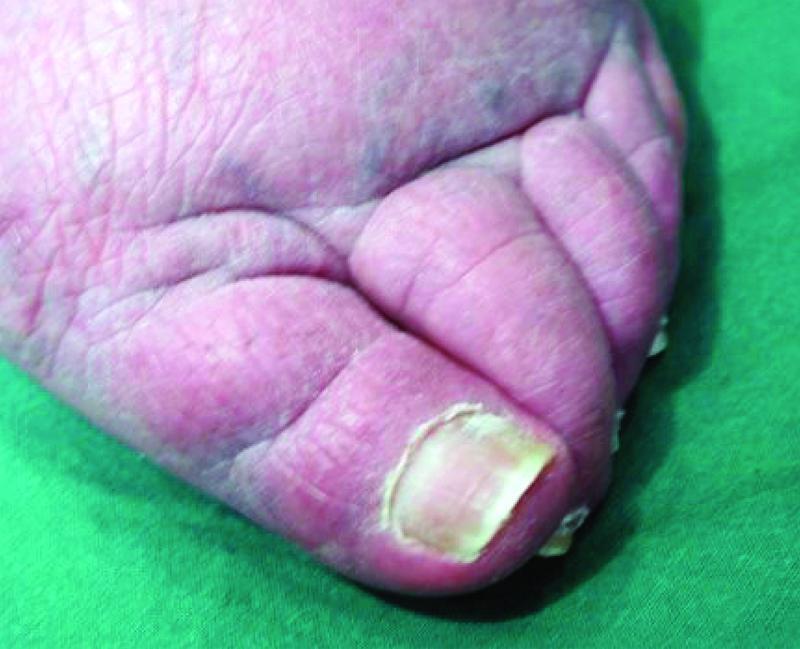
Pachyderma in secondary lymphedema. Lymphedema with skin thickening and fatty tissue proliferation leads to changes in the shape of the skin mantle, here: Box toes, transverse furrows, and Kaposi–Stemmer sign positive.

Even if the operation is performed with the inclusion of the affected phalanges, the pachyderma typical of lymphedema cannot be changed by “lymphological liposculpture” 10 12 42 54 55 56 57 58 ( Fig. 7 ).

Postoperative care has been developed as “AMLD” to drain the lymphatic vessels and improve transport performance:

- Postoperative 24-hour inpatient admission
- Full mobilization and discharge on the first postoperative day after 30 minutes of intermittent pneumatic compression therapy

- Treatment with a compression stocking plus overlying bandaging from peripheral to central

The retromalleolar area, called “Bisgaard Kulisse,” is compressed with additional padding. Further postoperative care is provided on an outpatient basis by the patient's physiotherapist on site:

- Fortnightly, therapy with daily MLD as a 60-minute full treatment of both arms and legs with accentuation of MLD on the operated limb (AMLD) and compression stocking class 2 plus additional pelottes and bandaging to increase local pressure

For another 2 weeks, gradual reduction of AMLD:

- MLD thrice a week
- Daily 12-hour compression by stockings, and, if necessary, pads

**Fig. 8:**
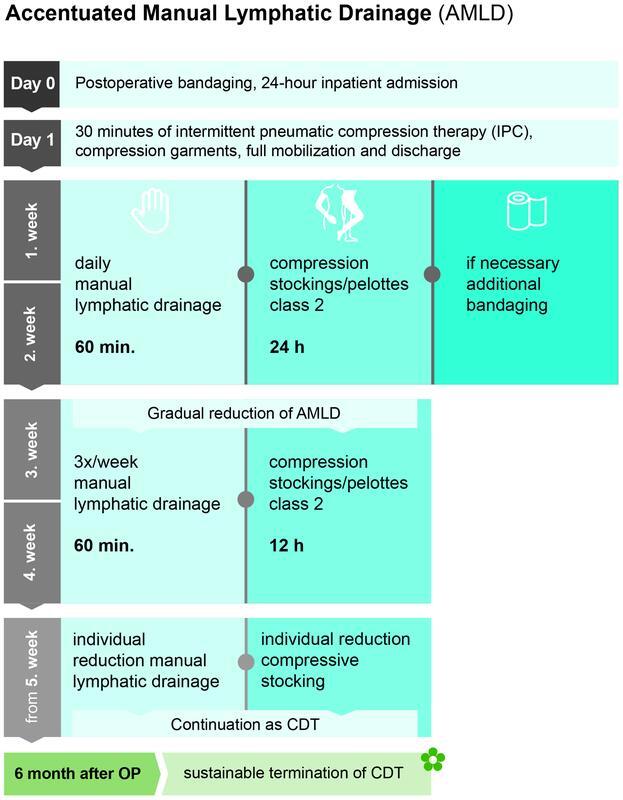
Time frame of accentuated manual lymphatic drainage in lymphatic liposculpture of secondary lymphedema. CDT, complex decongestive therapy.

From the fifth week, MLD and compression are reduced individually. The goal is the sustainable discontinuation of CDT after 6 months 9 42 53 ( Fig. 8 ).

Postoperative medical treatment includes 14 days of oral antibiotics with cefuroxime 3 × 500/d as infection prophylaxis and 3 days of thromboprophylaxis with low molecular weight heparin, e.g., enoxaparin 40 mg subcutaneously. 59

## Results

The results of treatment by “lymphological liposculpture” after up to 15 years were evaluated in 65 patients. The following cancers had been initially treated and preceded the development of secondary lymphedema of the extremities: breast carcinoma ( *n* = 24), cervical carcinoma ( *n* = 19), uterine carcinoma ( *n* = 11), ovarian carcinoma ( *n* = 4), vulvar carcinoma ( *n* = 2), and as further nongynecological entities melanoma ( *n* = 3), urethral carcinoma ( *n* = 1), and non-Hodgkin lymphoma ( *n* = 1).

The patients were 43 (mean [M]: 43; standard deviation [SD]: 11; SD for a sample [S]: 14–67) years old at the time of cancer.

Between the oncological intervention and the diagnosis of secondary lymphedema, there were approximately 4 years (M: 3.81; SD: 5.02; S: 0–22); between the lymphological diagnosis and the “lymphological liposculpture,” there were further 6 years of conservative therapy (M: 6.14; SD: 6.45; S: 0–37). The patients were 52 (M: 52.94; SD: 11.04; S: 0–75) years old at the time of “lymphological liposculpture.”

### Lateral Alignment and Volume Enhancement

In the study, the postoperative changes in volumes were measured perometrically in 42 patients ( Table 2 ).

**Table 2:** Perometer measurement

Patient No.	Extremity	Volume left in mL	Volume right in mL	Difference healthy side in mL	% Difference volume opposite side	Deviation healthy side in %
1	Legs	8,905	8,502	403	104.74	4.74
2	Legs	7,700	7,632	68	100.89	0.89
3	Legs	7,677	7,620	57	100.74	0.74
4	Arms	2,510	2,500	10	100.40	0.40
5	Legs	7,101	7,682	581	108.18	8.18
6	Legs	7,724	7,800	76	100.98	0.98
7	Arms	2,510	2,400	110	104.58	4.58
8	Legs	8,400	8,307	93	101.11	1.11
9	Arms	4,000	4,200	200	105.00	5.00
10	Arms	3,855	4,335	480	112.45	12.45
11	Arms	2,736	3,100	364	113.30	13.30
12	Legs	8,700	8,462	238	97.26	−2.74
13	Legs	6,900	7,000	100	101.44	1.44
14	Legs	6820	6800	−20	99.70	−0.30
15	Legs					
16	Legs	9,200	8,215	985	111.99	11.99
17	Legs	8,404	8,275	129	101.56	1.56
18	Legs	7,566	7,376	190	102.58	2.58
19	Arms					
20	Legs	5,941	6,632	691	111.63	11.63
21	Legs	8,860	8,000	860	110.75	10.75
22	Arms	4,508	4,485	23	100.51	0.51
23	Arms					
24	Arms					
25	Legs					
26	Legs	7,212	6,730	482	107.16	7.16
27	Arms	2,547	2,706	159	106.24	6.24
28	Legs	7,724	7,770	46	100.60	0.60
29	Arms	3,142	3,325	183	105.60	5.60
30	Arms					
31	Arms	3,325	3,142	183	105.82	5.82
32	Legs	8,566	8,376	190	102.27	2.27
33	Arms					
34	Legs					
35	Arms					
36	Legs	7,549	7,860	311	104.12	4.12
37	Arms	2,904	2,570	234	108.76	8.76
38	Arms					
39	Legs					
40	Legs	9,721	9,995	274	102.81	2.81
41	Legs					
42	Legs					
43	Legs	11,100	10,562	538	105.09	5.09
44	Arms					
45	Legs	7,055	7,900	845	103.22	3.22
46	Arms					
47	Legs	7,653	7,364	289	103.92	3.92
48	Legs					
49	Arms					
50	Arms	3,106	3,134	−28	99.11	−0.89
51	Legs	8,996	9,040	−44	99.51	−0.49
52	Legs					
53	Legs					
54	Legs					
55	Legs					
56	Legs					
57	Legs					
58	Arms	3,743	3,318	425	112.81	12.81
59	Arms	3,400	3,620	220	106.47	6.47
60	Arms	3,712	3,017	695	123.04	23.04
61	Legs	8,273	8,304	31	100.37	0.37
62	Legs	8,300	8,210	110	101.09	1.09
63	Legs	7,965	7,646	319	104.17	4.17
64	Legs	8,566	8,376	190	102.27	2.27
65	Arms	3,100	2,800	300	110.71	10.71

Notes: Comparison of operated and healthy limb. Yellow: affected limb; green: ± deviation from healthy limb in %; gray: patient did not show up for the measurement.

In “lymphological liposculpture” of the arm, an average of 1,571 mL (M: 1,571; SD: 579; S: 600–3,500) aspirate was obtained. In the leg operations, an average of 5,060 mL (M: 5,060.70; SD: 2,807.12; S: 1,100–12,900) of tissue was aspirated. The aspirate includes the total amount per operation. Postoperative differences between +985 and −44 mL were measured to the respective healthy side over all extremities. The average deviation for arms and legs was + 4.42% (S: +23.04 to −2.74%).

### Lymphedema of the Arm

In total, the data of 15 patients with arm lymphedema could be analyzed. The volume of the healthy side was on average 3,143 mL (95% confidence interval [CI]: 2,812.69, 3,473.44).

When the healthy side was the right arm ( *n* = 9), the mean volume was 3,051 mL (95% CI: 2,574.87, 3,528.69). If the healthy side was the left arm ( *n* = 6), the average volume was 3,280 mL (95% CI: 2,665.26, 3,894.74).

The volume of the operated side was on average 3,380 mL (95% CI: 3,030.37, 3,730.16). If the operated side was the left arm ( *n* = 6), the average volume was 3,268 mL (95% CI: 2,775.00, 3,762.33). If the operated side was the right arm ( *n* = 9), the mean volume was 3,547 mL (95% CI: 2,882.44, 4,212.89).

The volume of the operated side was on average 7.67% larger than on the healthy side (95% CI: 4.26, 11.08).

The volume of the operated arm was significantly larger than that of the healthy side ( *t* (14) = 4.76, *p* = 0.000). The analysis of variance (ANOVA) showed no significant side differences in the volumes of the healthy side ( *F* (1,13) = 0.51, *p* = 0.488) or the volumes of the operated side ( *F* (1,13) = 0.69, *p* = 0.422).

### Lymphedema of the Leg

In total, the data of 27 patients with a condition following lymphedema of the lower limb could be evaluated. The volume of the healthy leg was on average 7,915 mL (95% CI: 7,540.42, 8,289.88).

When the healthy side was the right leg ( *n* = 16), the mean volume was 8,137 mL (95% CI: 7,685.48, 8589.64). If the healthy side was the left leg ( *n* = 11), the average volume was 7,591 mL (95% CI: 6,900.26, 8,283.01).

The volume of the operated leg averaged 8,194 mL (95% CI: 7,817.76, 8,570.79). As for the operated side the left leg ( *n* = 16), the average volume was 8,441 mL (95% CI: 7,952.42, 8,931.33). As for the operated side the right leg ( *n* = 11), the mean volume was 7,834 mL (95% CI: 7,215.03, 8,456.5).

The volume of the operated leg was on average 3.66% larger than on the healthy side (95% CI: 2.04, 5.29).

The volume of the operated leg was significantly larger than that of the healthy leg ( *F* (1,26) = 4.72, *p* = 0.000). The ANOVA showed no significant side differences in the volumes of the healthy side ( *F* (1,25) = 2.27, *p* = 0.144) or the volumes of the operated side ( *F* (1,25) = 2.85, *p* = 0.104).

**Fig. 9:**
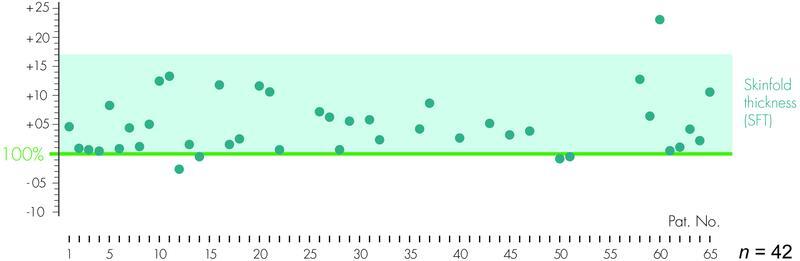
Postoperative perometric measurements to record side alignment. The volume of the unaffected side is set to 100% as the target value for comparison. The postoperative volume difference of the 42 patients measured is presented as a deviation in % from this 0-line (± ). Note the range of skinfold thickness (SFT) in %.

The healthy limb was evaluated with 100% and the difference was determined as a measure for volume approximation and side alignment in %. Values close to zero result in equality of the sides, oversuction results in a negative value, positive values in a circumferential difference in favor of the operated side ( Fig. 9 ).

### Therapy Abstinence and Reduction of Complex Decongestive Therapy

In 38 patients (58.46%), further CDT could be dispensed with at the latest 6 months after “lymphological liposculpture.” A considerable reduction of CDT was reported by 22 patients (33.82%) in different ways. In 5 of 65 (7.69%) patients, conservative treatment could not be reduced after the surgical intervention ( Table 3 ).

**Table 3:** Pre- and postoperative conservative therapy ( *n* = 65)

Preoperative CDT frequency		
1× wk	7	10.76%
2× wk	43	66.15%
3× wk	15	23.07%
Postoperative conservative therapy		
No more CDT	38	58.46%
CDT 1× wk	11	16.92%
CDT every 2 wk	3	4.61%
CDT every 4 wk	1	1.53%
Only MLD/no stocking	2	3.07%
Every 2 wk only MLD	5	7.69%
Continued thrice a week CDT	5	7.69%

Abbreviations: CDT, complex decongestive therapy; MLD, manual lymphatic drainage.

**Fig. 10:**
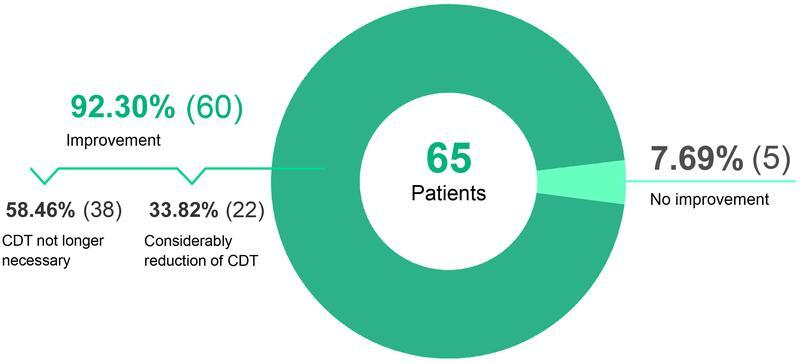
Complex decongestive therapy (CDT) after “lymphological liposculpture”.

The goal of “lymphological liposculpture” in secondary lymphedema to achieve a therapy abstinence of CDT or to reduce the need for conservative treatment to 20% or less of the initial value was achieved in 92.30% ( *n* = 60) of the patients ( Fig. 10 ).

### Quality of Life

**Fig. 11:**
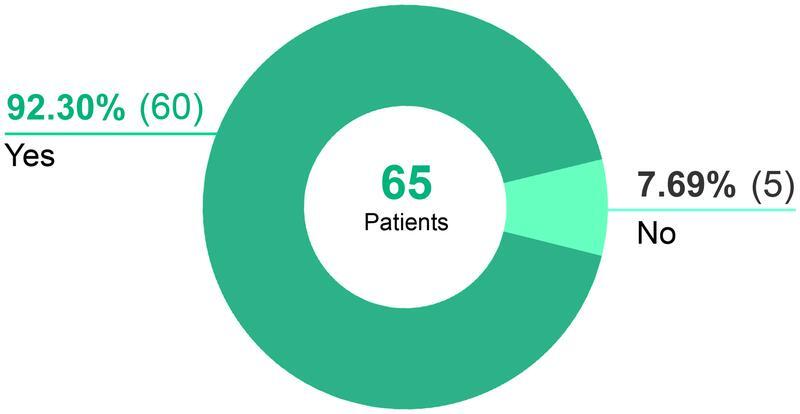
Has your quality of life improved as a result of the operation.

Improving the quality of life is considered one of the three goals of the surgical method. “Has your quality of life improved as a result of the operation?” Sixty women (92.30%) answered with “Yes” and five women (7.69%) with “No” ( Fig. 11 ).

**Fig. 12:**
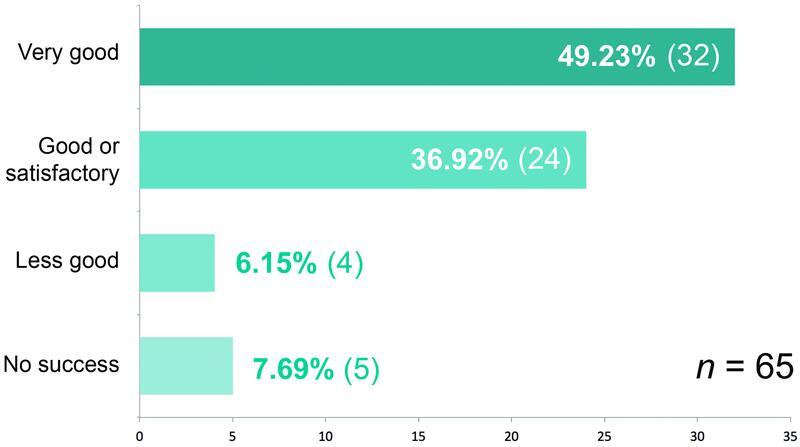
Assessment of the success of the treatment.

In the evaluation of the success of the treatment, 32 of the 65 women (49.23%) rated it as “very good,” 24 (36.92%) as “good” or “satisfactory.” Four (6.15%) patients rated the treatment as “less good” and five (7.69%) as “no success” ( Fig. 12 ).

**Fig. 13:**
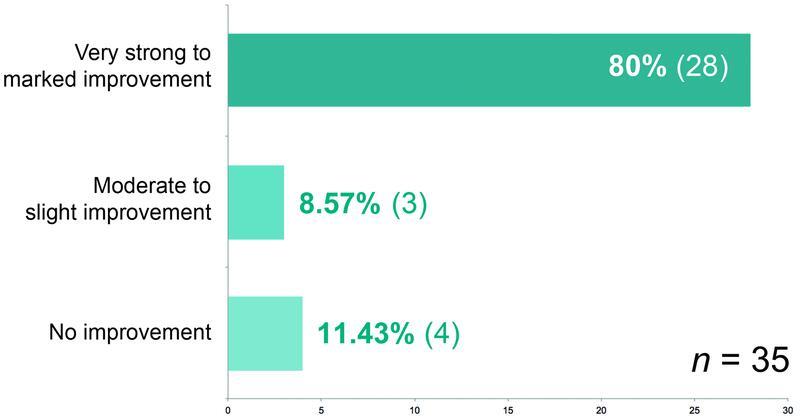
How has your limitation of movement improved since your last operation?

Restricted mobility is a typical consequence of lymphedema, reported by 35 (53.85%) of the 65 patients. We asked about improvement of these limitations. Twenty-eight (80%) of the women reported a “very strong” to “marked” improvement in the limitation of movement; another three (8.57%) patients felt a “moderate” to “slight” improvement. In four (11.43%) patients who had surgery, the limitation of movement did not improve 11 19 20 ( Fig. 13 ).

**Fig. 14:**
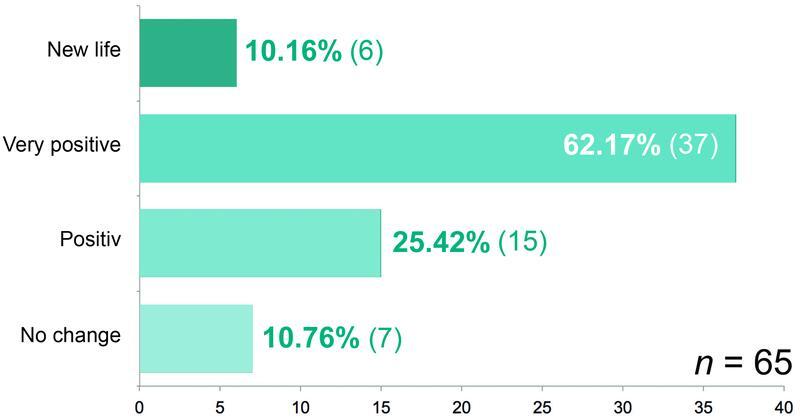
How has your self-esteem changed after your last surgery?

We asked about the self-esteem of our patients before the lymphedema operation and whether it had changed after the “lymphological liposculpture.” While 41 (63.07%) of the patients stated before the operation that they had suffered a loss of self-esteem due to the lymphedema and 24 (36.92%) did not feel this way, the assessment of self-esteem changed after the operation. Now, 58 (89.23%) of the patients perceived a positive effect of the treatment of the disease on their self-esteem. Six (10.16%) noted “new life,” 37 (62.17%) “very positive,” and 15 (25.42%) “positive” ( Fig. 14 ). Sixty out of 65 (92.31%) would recommend this surgical therapy for the treatment of secondary lymphedema to their best friend.

## Discussion

Every year, more than 30,000 patients in Germany develop secondary lymphedema after lymphadenectomy for breast carcinoma and gynecological tumors. For these cancers, an incidence of secondary lymphedema of 30.5% on average can be assumed. 3 4 5 6 7 8 24 27 40 60 61 For breast carcinoma, the risk depends on the tumor itself, the different therapy measures and the individual anatomical factors of the patient. 60 61 It remains unclear why not all breast cancer patients treated in the same way develop lymphedema. The development of lymphedema obviously does not correlate with the quality of the surgical intervention. One reason may be the individual variance in the number of lymph nodes, and thus the number of lymph channels. The more lymph nodes there are per lymph node station, the lower the risk that the removal of only one or a few lymph nodes (Sentinel nodectomy) will lead to lymphedema.

Independent risk factors, on the other hand, are:

- Increased body mass index (BMI),
- An advanced tumor stage with lymph node involvement, with a peritumoral lymphovascular invasion and extranodal tumor growth,
- Surgical measures as well as radiotherapy in the area of the lymph drainage area,
- Adjuvant chemotherapy,
- Infections in the operated area,
- An erysipelas or recurrences of erysipelas after completed therapy.

Patients with lymphedema can be treated either conservatively with lymphatic drainage and compression (CDT) for life or surgically. 11 19

There are three goals for surgical intervention for lymphedema:

- The elimination of the lateral difference by equalizing the volume of the extremities is aimed at.
- The burden of MLD and compression should be significantly reduced. Ideally, CDT and the stigmatizing compression stocking on the arm or leg can be dispensed with.

- A considerable improvement in the quality of life is intended.

Surgical treatment options for lymphedema include resection of subcutaneous tissue or lymphatic vascular surgery performed as LVA, LVT, LVB, or VLNT. 28 43 44 45 46 47 62

### How Are Reconstruction and Resection to Be Evaluated. Is a Procedure Algorithm Already Possible?

The evolution of the paradigm shift in the treatment of secondary lymphedema is orchestrated by the discussion of whether resection or reconstructive surgery alone or the combination of surgical procedures will achieve the goal of aligning the affected limb with the opposite side and freeing the patient from any further conservative therapy. Recent publications show encouraging results of microsurgery.

LVA and VLNT can be successfully omitted when lymphatic interposition flap transfer (LIFT) is used for soft tissue defects of the extremities, preventing the development of secondary lymphedema. 38

Similarly, treatment of primary and secondary upper or lower extremity lymphedema with VLNT, whether the flaps were jejunal mesenteric, inguinal, lateral thoracic, omental, or right gastroepiploic and submental, results in progressive, significant reductions in limb volume, extracellular fluid, and cellulitis episodes. Patients also reported positive outcomes 6 months after surgery compared with maximal conservative therapy alone. Unfortunately, it is not reported whether of the 134 patients further conservative therapy could be avoided altogether and how stable the outcome was over subsequent years. 32

Visconti reports that different concepts of lymphatic transfer—VLNT, lymphatic vessel transfer (LIFT), lymphatic system transfer—are used today, so that the different reconstructive needs of lymphedema patients can be met. The follow-up of the 45 was 12 months. 30 Compression garments could not be discontinued in this study. Twelve patients (26%) had more stable results with physical treatment, and in only 1 case (3.3%) could compression be reduced by one class. However, in none of the 30 patients could further conservative therapy be completely abandoned. Encouragingly, no posttraumatic lymphedema developed in any of the prophylactic LIFTs.

In contrast to this work by Visconti, after the lymphatic liposculpture procedure presented in this paper, 38 patients were able to completely forgo further conservative therapy and 22 patients were able to significantly forgo further conservative therapy, resulting in a total of 60 of the 65 patients benefiting from resection.

Even though functioning lymphatic vessels are no longer detectable in advanced-stage lymphedema patients by lymphatic scintigraphy and indocyanine green lymphography, ultrahigh-frequency ultrasound allows the identification of such lymphatic vessels relevant to LVA. This methodological advancement is significant for expanding the indication for patients with stage III ISL. 11 29 Unfortunately, no further patient outcome data are provided in this paper. The use of ultrahigh-frequency ultrasound in clinical routine for preoperative marking of veins and lymphatic vessels allows on the one hand to significantly shorten the operation time and on the other hand to perform minimally invasive LVA surgery on patients with massive lymphatic fibrosis and thick dermis (up to 5 mm). 63

The combined use of duplex ultrasound and magnetic resonance lymphangiography to identify functional lymphatic vessels allows the creation of functional lymphovenous anastomoses in the lower extremities even in ISL stage II and III. 33 In this study, deep lymphovenous anastomoses were created. Mean limb volume progressively decreased postoperatively up to 15.5% at 1 year. Among patients with unilateral lymphedema, 32.4% had less than 10% excess volume postoperatively compared with the contralateral side, whereas 20.5% had more than 20% excess volume. Unfortunately, no further patient outcome data are provided. These volume reductions, as well as the significant improvement in the incidence of erysipelas, are comparable to the results of volume improvement after resection by lymphatic liposculpture. Including the intrinsic thickening of the cutis, we measured average differences of 7.67% on the arm and 3.66% on the leg compared with the healthy side.

Resection and reconstructive microsurgery are not in competition. However, this paper is concerned with the evaluation of resection as the sole procedure. The goal of treatment should always be to equalize volumes while taking into account SFT and foregoing further lifelong CDT. Different follow-up time, different weighting of outcomes, different postoperative therapy regimens, strongly differing patient numbers, and missing data on surgery times do not allow the comparison of studies focusing on the effects of reconstructive and resection procedures.

Thus, despite all the positive results of reconstructive procedures, there is still a lack of an international treatment algorithm “operative lymphology” to “triage” the right patients for the best method. The outcomes for the patients—no more conservative therapy, lateral alignment of the extremities taking into account the SFT, improvement of the quality of life—should be the priority here. The general use of the validated Lymphedema Quality of Life Questionnaire (LYMQOL) would rather allow the comparability of surgical methods. Valid long-term results can also be collected in this way.

Since 1997, the “dry technique” by Brorson and the “wet technique” “lymphological liposculpture” by Cornely have been available as resection methods. 9 34 35 36 37 42 48 49 50 53 64

Brorson postulates that improvement in treatment is possible through early surgical intervention. He focuses on the removal of the lateral difference. Continuous, lifelong compression therapy is still necessary after surgery to maintain surgical tissue reduction and control of edematization. 35 48 65 His method does not allow for the waiving of further lifelong postoperative compression. 35 Brorson's “dry liposuction” is free of major surgical complications, although blood transfusions may be required.

Contrary to Brorson's basic assumption, the lymphatic vessels are largely undamaged in secondary lymphedema after oncological treatment because secondary lymphedema does not damage the lymphatic vessels a priori. 10 12 Sclerosis-related lumen narrowing or wall thickening may occasionally occur as a consequence of lymphedema, but this is not necessarily the case. 10 12 The outflow obstruction and the consecutive growth of solid tissue are initiated by the lymphadenectomy and other causes. 3 4 5 6 7 8 24 27 42 60 61 65

“Lymphological liposculpture” as a “wet technique” under TLA was originally used in 1997 without the addition of hyaluronidase for the treatment of nononcological “lipedema” patients (LiDo) and marks the paradigm shift in the treatment of this disease from exclusively conservative to surgical therapy. 36 42

The removal of subcutaneous tissue gel-like edematized by PG and GAG in primary and secondary lymphedema was a new and special challenge. TLA was modified by the addition of the enzyme hyaluronidase (TLA + H). It's now lytic effect on the central HA filament of the PG supports the preparation of the subcutaneous matrix in lymphedema. 37 This use of hyaluronidase is an off-label use. However, even with the use of up to 11,000 IU of hyaluronidase per surgery, there were no side effects. Neither redness, pain, nausea, or vomiting nor pectanginal complaints or dizziness, shortness of breath, eczema, urticaria, pseudoallergic reactions in the area of the face, lips, neck, ears, arms, or legs occurred. 37

Used since 2005 as a resection procedure for primary and secondary lymphedema in TLA + H, the “lymphological liposculpture” is particularly successful as a further development of liposuction from the “dry technique” with the Löfgren cuff in bloodlessness to the “wet technique,” as postoperative blood transfusions and often lifelong CPD can be dispensed with. 36 37 42 50 65 Adverse effects such as infections or seromas occurred very rarely during surgery, with a frequency of <1%. Other complications such as necrosis or thrombosis, especially hemoglobin loss with the need for blood transfusions, as known from the “dry technique” 35 48 , did not occur with liposculpture using the “wet technique.” 54 59

The advantage of the resecting procedures in contrast to the vascular reconstructive procedures such as LVA and VLNT is the low effort in preparation, the manageable effort with short operating theater occupancy time and the short treatment time of the patient. 37 50 Excluding the TLA + H infused into the tissue in the operating theater and the subsequent preparation time in the anesthesiologic recovery room (holding area), the duration of surgery for “lymphological liposculpture” requires 30 minutes for a lymphedema arm, 40 minutes for a lymphedema leg, and 60 minutes for lymphedema of both legs. If success is not sufficient after 6 months of postoperative AMLD, lymphatic vessel operations can be performed in a further step, preferably as deep lymphatic anastomoses (LVA). 28 29 31 33 66 Depending on the degree of sclerosis of the lymphatic vessels, a VLNT may also be indicated. 11 27 28 29 30 32 55 62 67 In this study, no LVAs or other vascular reconstructive procedures were performed in combination with resection as lymphatic liposculpture.

### Cutis Thickening, Lateral Alignment, Movement Restriction, and Therapy Abstinence

In the literature, information on standard volumes of arms or legs, on the lateral difference of the two upper and lower extremities or on SDs at extremities cannot be found. Only Kasseroller gives the volume for the upper extremity in women with lymphedema after breast carcinoma. 10 In an examination of 587 patients with a positive history of breast cancer and 58 healthy women, taking into account handedness, he calculated an average volume of 2,513 mL (scatter: 1,701–4,009) for the healthy arm of the woman using Kuhnke's method. 10 68 By definition, the increase in volume of the lymphedema affected limb in stage II is reversible by CDT and irreversible from stage III. 11 19 56 However, already from stage I, the intracutaneous disease lymphedema is accompanied by obligatory cutaneous thickening. 12 57 Resection of subcutaneous tissue reduces lymphedema volume (LOEV) significantly better than CDT alone but does not change SFT. The increase of SFT in diseased patients is not related to the increase in volume of the limb. Even with only a small increase in volume, the SFT is significantly altered. In the healthy arm, the cutis thickness is 3% volume-forming. In severe lymphedema with an LOEV > 150%, SFT of 7.6 to 10.5 mm are measured posttherapeutically. 10 11 This cutaneous thickening contributes up to 17% of the volume. Moderate LOEV of 125 to 150% have SFT of 5.8 to 7.2 mm; the volume contribution of cutaneous thickening is up to 13.5%; mild LOEV of <125% have SFT of 4.3 to 5.9 mm on average with a volume contribution of 11.7% (result of the statistical analysis of the volumes separated for arms and legs). A plus in the lateral comparison of the extremities due to the skin thickness alone is typical of the disease. Since the SFT cannot be changed by suction, a complete volume equalization of the extremities after disease by secondary lymphedema could only be achieved by oversuction. 10 12 54 57 58 We achieved average lateral differences of 7.67% on the arm and 3.66% on the leg compared with the healthy side, which are also due to the immanent thickening of the cutis.

The volumes of the extremities are also dependent on changes in BMI. However, we could not BMI match the perometer data in this follow-up of up to 15 years because of the temporal dimension.

The solid tissue proliferation on the extremity is not only cosmetic but also orthopaedically relevant due to its weight. Movement restrictions due to lymphedema are regular and affected 35 of the 65 patients in our study group. 11 20 24 By removing the mass gain, the improvement of the movement restriction was achieved in 31 of the 35 patients in varying degrees.

Volume reduction also has infection-protective effects. Eleven patients (16.92%) reported up to 12 recurrent erysipelas per year before surgery. After surgery, no patient had recurrent erysipelas during the observation period.

We achieved alignment with the healthy limb in all patients. Regarding the abstinence of conservative treatments, 38 patients stated that they were completely free of symptoms and no longer needed further conservative therapy. Twenty-two patients were able to limit conservative therapy considerably, so that a total of 60 of the 65 patients benefited from resection.

The application of the surgical procedure “lymphological liposculpture” with its two-part approach of surgery and conservative follow-up treatment leads to a curative and sustainable volume adjustment and to the reduction or abandonment of CDT. In what is usually a one-stage procedure, the hypertrophic subcutaneous tissue can be removed with an excellent clinical result.

### Quality of Life

For 60 patients in the study, the quality of life also improved considerably by reducing or avoiding any CDT after the operation. The majority of patients would recommend “lymphological liposculpture” to their best friend as a surgical treatment for lymphedema. In addition to the achieved therapy abstinence and the improvement of the movement restriction, the positive influence on the item “change in self-esteem” through the lymphedema operation certainly contributes to this. After the operation, 43 patients noted a positive effect.

### Limitations of the Study

We were not able to personally follow up all patients for the preparation of the study. However, the 100% response rate to the questionnaire demonstrates the patients' interest in reporting on the course of their disease. Additional telephone enquiries with all patients ensured that the information was correct. However, the perometric measurements in the study center on site could only be performed for 42 patients. There are no standard measures for arms and legs, volume of extremities, or their SD in the literature. The changes in the cutis caused by the secondary lymphedema could only be recorded approximately.

Since the aim of this study was to investigate the equalization of the lateral difference, the collection of pre- and postoperative volume measurements to document the success of the surgery itself was omitted. For further investigation of the effects of the surgical method, the measurement protocol has now been changed, and the preoperative findings of all new patients are also recorded perometrically. The use of the validated LYMQOL will allow comparability among surgical methods. Valid long-term results can also be collected in this way.

The primary goal of surgical lymphology is to achieve permanent freedom from symptoms and to end the conservative therapy of lymphedema. The procedure of “lymphological liposculpture” is suitable for secondary lymphedema to significantly reduce the need for lifelong implementation of CDT; in a predominant number of patients, further conservative therapies can be completely dispensed with. The improvement of movement restriction, the stocking, and the associated stigmatization can be dispensed with. The adaptation of the extremities, the cosmetic regeneration, and the improvement of the quality of life can be achieved. In a next step, if necessary, additive LVA could further optimize the results.

We also recommend resection of the subcutaneous tissue in cases of longstanding secondary lymphedema stage II and III using the “lymphological liposculpture” procedure to equalize the volume of the extremities, consecutively reduce the CPD and improve the quality of life.
